# The assessment of a novel lower body resistance garment as a mechanism to increase the training stimulus during running: a randomised cross-over study

**DOI:** 10.1186/s13102-022-00455-9

**Published:** 2022-04-08

**Authors:** Samantha M. Hoffmann, Isaiah Di Domenico, Paul K. Collins

**Affiliations:** 1 Centre for Sport Research (CSR), School of Exercise and Nutrition Sciences, Deakin University, Geelong, VIC Australia; 2 Centre for Sport Research (CSR), School of Engineering, Deakin University, Geelong, VIC Australia

**Keywords:** Wearable resistance, Limb loading, Exercise physiology

## Abstract

**Background:**

This study examined the physiological and perceived impact of wearing a novel lower body resistance garment during exercise and recovery.

**Methods:**

Using a randomised cross-over design, 15 recreationally-active males performed 2 × 10-min steady-state runs followed by a 10-min passive recovery with concomitant monitoring of oxygen consumption (V̇O_2_), heart rate (HR) and rating of perceived exertion (RPE; exercise portion only), wearing either the resistance garment (experimental) or running shorts (control).

**Results:**

During exercise, there was a trend for V̇O_2_ and RPE to be higher (4.5% and 7.7% respectively) in experimental than control (V̇O_2_: *r* = 0.24, *p* > 0.05; RPE: *r* = 0.32, *p* > 0.05) and for HR to be lower (− 0.4%, *r* = − 0.05, *p* > 0.05). During recovery, V̇O_2_ and HR tended to be lower (4.7% and 4.3% respectively) in experimental than control (V̇O_2_: *r* = − 0.32, *p* > 0.05; HR: *r* = − 0.27, *p* > 0.05).

**Conclusions:**

Though effects were trivial to small, and not statistically significant, these findings provide proof of concept and suggest that this garment design may increase the training stimulus during running and aid post-exercise recovery.

## Background

Progressive overload ensures health and performance benefits are achievable and sustainable [1, 2]. There are many ways to apply progressive overload in exercise training with recreational and athletic populations [3]. Wearable resistance (WR) is a method whereby external loading is placed upon the body during exercise with the goal of increasing movement difficulty, therefore providing a greater training stimulus—all without causing undesirable movement patterns [4, 5]. A variety of WR loading orientations have been examined including trunk loading via weighted vests [6, 7], weighted pulley systems [6] and loaded back packs [8], and lower limb loading via thigh and foot weights sewn into clothing [9] and weighted ankle bands [10]. During exercise at sub-maximal intensities (6.4–14.4 km h^−1^), these loading orientations have shown considerable increases in oxygen consumption (V̇O_2_) [6, 7, 9, 10], heart rate (HR) [7–10], and perceived exertion [8] during exercise. However, negative side effects including altered running mechanics have been reported when weighted vests were worn [6] and discomfort has been perceived with the use of weighted ankle bands [10] and loaded back packs [8].

Previous research has examined varying magnitudes of applied resistance in WR apparel on both acute and chronic responses to exercise. Low levels of resistance (1–5% body mass; BM) in the form of compression shorts increased HR (0.4–2.9%) and V̇O_2_ (1.7–8.1%) in endurance athletes during 8 min of steady-state running [11]. Conversely, with higher levels of resistance (5–10% BM) in the form of weighted vests, endurance athletes experienced no significant increase in V̇O_2_ during 12 min of steady-state running compared to control [6], and no significant increase in maximal oxygen consumption (V̇O_2_max) following four weeks of WR training when compared to control [12]. However, with a resistance of 10–20% BM using weighted vests, a significant increase in V̇O_2_ (10.8–16.8%) was shown in recreationally-active participants across all stages of an incremental walking test (2.0–4.0 mph) [7]. Further increases in resistance (20%, 30% and 40% BM) using weighted back packs also showed significant increases in HR (6.3–8.0%) and perceived exertion (16.7–40.7%) in recreationally-active participants during an 8 km walk at a self-selected pace [8]. These findings suggest that training status and/ or the magnitude of applied resistance may influence the capacity of WR to increase the physiological and perceived demand of exercise. As such, no common consensus has been reached regarding the optimal magnitude of applied resistance needed to sufficiently increase the physiological demand of exercise without inducing undesirable changes in movement patterns [4].

Recent research has focused on tightly fitted lower limb compression garments as a means of applying WR that is more comfortable for the wearer whilst avoiding undesirable impacts on movement mechanics. These designs include international standard compression materials that conform to the body’s natural shape ensuring comfort and avoiding range of motion restrictions [13]. The garments provide low levels of loading (1–5% BM) to the thigh via small, weighted pouches (100–200 g) attached to the garment at distal to proximal orientations [11]. Although the garment increased V̇O_2_, HR and perceived exertion during running, the weighted pouches were bulky and required participants to physically manipulate the garment to meet their desired level of resistance. Further research is warranted to explore more practical and effective ways in which WR can be used to increase training stimulus. No previous studies have reported the effect of WR on post-exercise recovery. This may be of practical significance if WR garments exhibit compression qualities. Despite inconclusive effects of compression garments on recovery indicators, there is some evidence for local blood flow augmentation [14], and therefore assessing WR during recovery is warranted. The aim of this study was to examine the physiological and perceptual responses to steady-state running and post-running recovery whilst wearing a novel lower body WR garment with 1–3% BM applied resistance compared to standard running shorts, in recreational exercisers.

## Methods

### Subjects

Subjects were recruited via convenience sampling through word of mouth and email advertisements sent to staff of the Health and Engineering faculties at Deakin University and to players from local amateur football clubs. Inclusion criteria were (a) males aged 18–35 years (inclusive), (b) free of injury and illness, (c) body mass index (BMI) < 30 kg.m^2^, (d) recreationally-active, defined as having completed a minimum of 60 min of moderate to vigorous intensity physical activity per week for the last six months. Fifteen recreationally-active men (mean ± SD, age: 24.5 ± 3.5 years; body mass: 75.5 ± 8.0 kg; height: 179.9 ± 8.8 cm) volunteered to participate. Ethics approval was obtained from the local Institutional Human Research Ethics Committee prior to participant recruitment. All subjects provided written informed consent and completed the Exercise and Sport Science Australia, Adult Pre-Exercise Screening System (ESSA-APSS) before participating. Completion of the ESSA-APSS confirmed participant eligibility.

### Procedures

The present study was conducted in two phases: (1) the design of the garment, and (2) human exercise trials.

#### Design phase

**Fig. 1:**
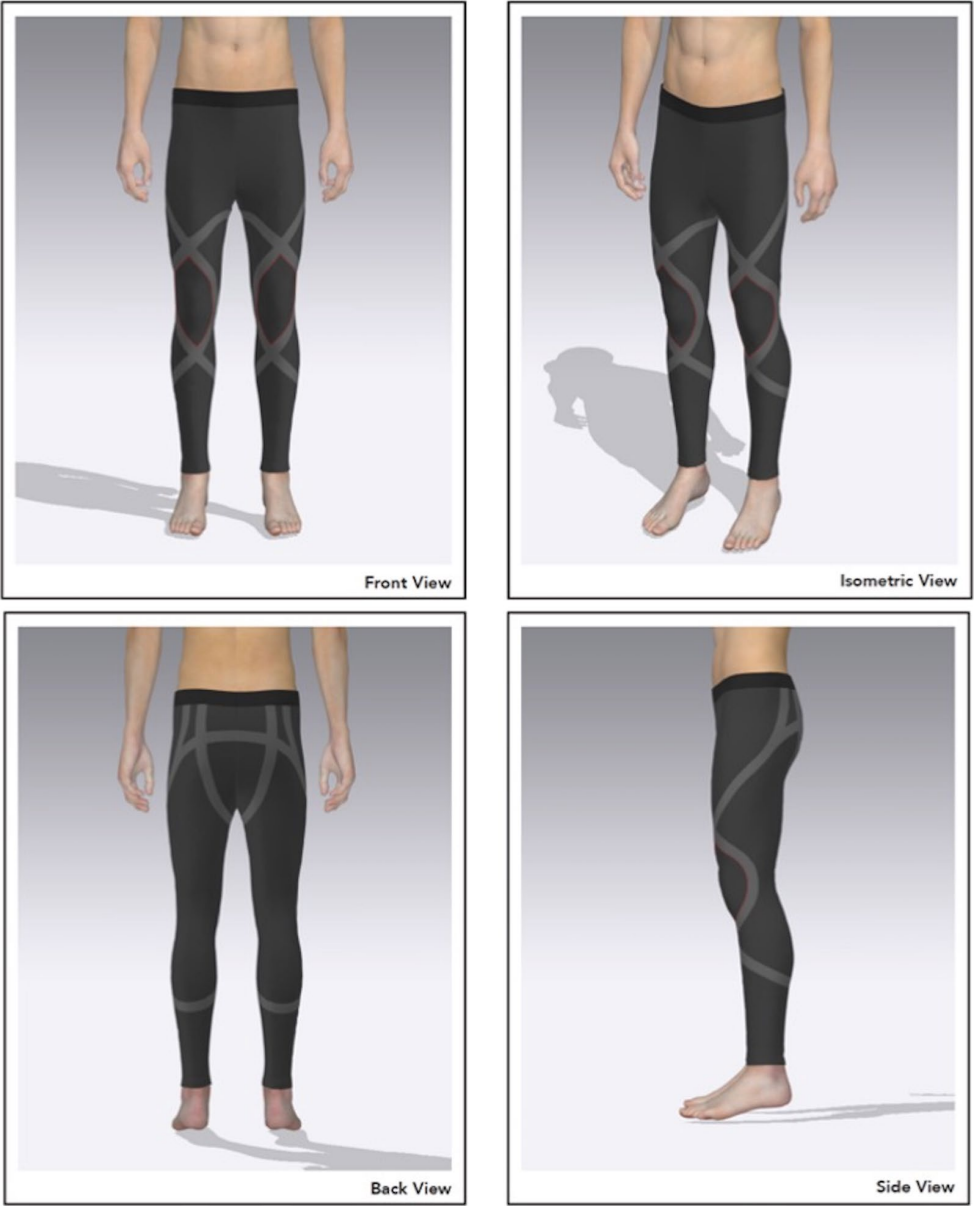
General design of the novel wearable resistance garment

The novel lower body resistance garment used in this study was comprised of ~ 92% polyamide and ~ 8% elastane wherein resistive bands were seamlessly and integrally formed in the fabric of the garment through 3D knitting techniques. As shown in Fig. 1, the ‘X’ shaped configurations applied 1–3% BM resistance to the wearer across various anatomical locations [15]. These included the hips and knees, and the musculature acting upon these joints including the quadriceps, hamstrings, glutes and lower leg muscles. This design differs to standard compression garments which apply general compressive forces across the entire garment rather than targeted resistance across specific areas. Due to the general design and materials used, the garment was intended to have a similar look and feel to a conventional compression garment that conformed smoothly to individual body shapes, ensuring comfort for the wearer during movement.

The design was partially developed by use of computer software (Abaqus V6.14) to investigate placement of the resistance-based zones as well as indicative elastic moduli of the knitted base materials. A Finite Element Analysis (FEA) model was created, based off a male in the 75th percentile for height (~ 178 cm) and weight (~ 75 kg). The FEA model compared joint reaction moments from the hip and knee and when compared to a non-WR garment, it was noted that 11.5% and 8.9% increases in reaction moments occurred at the hip and knee, respectively. Garment samples were produced and subsequently deconstructed with material samples tested on a 10 kN Instron load frame showing a difference in elastic modulus of the banded (6.98 ± 0.82 MPa) versus non-branded materials (3.13 ± 0.21 MPa). The FEA simulation in conjunction with material data, determined that the WR garment provided greater theoretical resistance against hip and knee rotation as compared to a traditional non-WR garment.

#### Exercise phase

A cross-over study design was used whereby all subjects served as their own control and reported to the Exercise Science Laboratory on two separate occasions separated by an average of 5 days. In a randomised order, subjects completed a steady-state running trial, once on each occasion, wearing either the novel lower body resistance garment (‘experimental trial’) or a pair of standard exercise shorts of their choosing (‘control trial’). Each subject completed their trials at a similar time of day under stable laboratory conditions (mean ± SD, temperature: 17.7 ± 1.2 °C; humidity: 43.3 ± 2.1%) on a motorized treadmill (150/50 LC, HP-Cosmos^®^, Nussdorf-Trainstein, Germany) at a gradient of 1% to reflect outdoor running demands [16]. For 24 h before each trial, participants were asked to (a) replicate their usual food and beverage consumption as closely as possible, (b) avoid consuming alcohol, (c) maintain a hydrated state, and (d) avoid vigorous or high-intensity exercise. Treadmill running speed was constant and set to one of two levels; ‘low’ (9.8 km h^−1^) or ‘high’ (11.9 km h^−1^), to account for cardiorespiratory fitness differences across subjects. Subjects who completed < 150 min of moderate-intensity physical activity per week were assigned to the ‘low’ running speed (*n* = *7*) and those who completed ≥ 150 min of moderate-intensity physical activity per week were assigned the ‘high’ running speed (*n* = *8*). This stratification criteria relates to the physical activity and exercise guidelines for Australian adults which specify a minimum of 150 min per week of moderate intensity physical activity for physical and mental health [17]. Both running speeds aimed to achieve an exercise intensity of 60% V̇O_2_max using the following equation: *V̇O*_2_ = *13.5* × *speed* − 8.5; a published equation for the relationship between V̇O_2_ and running speed [16]. V̇O_2_max for those classified as ‘low’ was based on normative data provided by the American College of Sports Medicine (ACSM) [18] for 20–29-year-old males (47 ml.kg^−1^.min^−1^). V̇O_2_max for those classified as ‘high’ was based on data from Australian Rules footballers (60 ml.kg^−1^.min^−1^) [19]. Both trials began with a 5-min warm up at a self-selected speed that was lower than the assigned running speed for the trial itself. Subjects then completed a 10-min run at their pre-assigned running speed, followed by a 10-min passive cool-down in a seated position on a regular chair with a back rest, and with their feet in contact with the ground. The duration of the run was informed by ACSM [18] exercise guidelines for healthy adults which encourages 30–60 min of moderate-to-vigorous exercise per day in bouts of 10 min or more. This duration was also deemed appropriate as exercise bouts of ~ 10 min are associated with improved health outcomes and similar health benefits to exercise bouts > 10 min [20, 21]. Additionally, previous research has implemented similar exercise durations to examine the impact of WR on physiological and perceptual outcomes [9, 11].

### Variables and measurements

Physiological responses were measured via HR and V̇O_2_. HR (beats per minutes; bpm) was measured using a chest strap HR monitor (Polar Electro Oy, KY, Finland). Gas exchange variables including V̇O_2_ (ml.kg^−1^.min^−1^) were measured using a metabolic measurement system (Metalyzer 3B, Cortex Medical, Leipzig, Germany) which was calibrated prior to each testing session with a known composition of ambient air (O_2_: 20.93% and CO_2_: 0.03%); this system has previously been shown as reliable (reliability coefficients for V̇O_2,_ V̇CO_2_ and V̇E = 0.969, 0.964 and 0.953, respectively) [22].

Participants wore a size-appropriate face mask connected to the metabolic measurement system via an air flow line, allowing them to breathe freely through their mouth and nose. HR was measured continuously and V̇O_2_ data were recorded breath-by-breath for the duration of the 10-min run and 10-min passive cool-down. HR steady-state has been reported to occur within 2–4 min of exercise at constant sub-maximal work rates [23, 24]. Thus, to sufficiently achieve the aims of this study, the final five minutes of the running test were analysed, and the first five minutes of the cool-down were examined for consistency purposes. Perceptual responses were measured via rate of perceived exertion (RPE) using Borg’s 6–20 RPE scale [25] every 2.5 min during the running portion of the test. HR and V̇O_2_ data were smoothed to 30 s intervals to de-emphasise recorded breath-to-breath variations. HR monitor malfunctions were experienced during five separate 30-s intervals resulting in obvious errors; these data points were subsequently removed before analysis.

### Statistical analysis

All statistical analyses were completed using IBM SPSS Statistics for Windows (version 25.0; IBM Corp., Armonk, N.Y., USA). Assumption testing showed not all variables met parametric assumptions for normality or variance. Given this outcome, and the size of the data sample examined, descriptive statistics (median and interquartile range) and percentage changes were calculated for all variables during the run and cool-down portions of the trials. Individual V̇O_2_ and HR responses during the run and cool-down were also examined to understand inter-individual variability. Wilcoxon Signed-Rank Tests were used to compare descriptive statistics between control and experimental trials for all variables, with effect sizes calculated using the following equation;

$$r = \frac{Z}{\surd N}$$

Key: r: Effect size, Z: Standardized z-score, N: Total number of observations.

Effect sizes were categorised using the following scale; < 0.2 = trivial effect, 0.2–0.5 = small effect, 0.5–0.8 = moderate effect, > 0.8 = large effect [26]. Friedman’s test was used to identify significant differences between control and experimental trials over time (trial x time). Statistical significance was set at *p* < 0.05.

## Results

### Collective responses during exercise and passive recovery

**Fig. 2:**
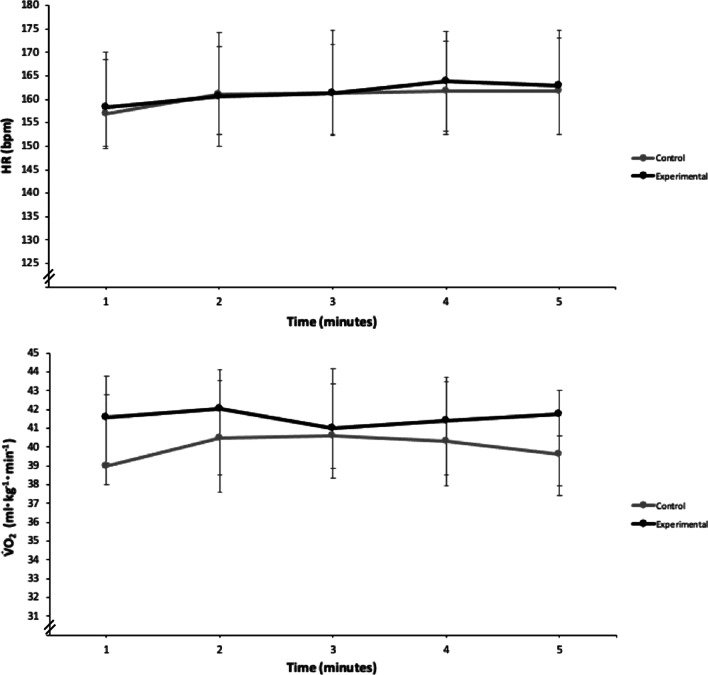
Physiological responses during five minutes steady-state running in control and experimental trials (n = 15). Data presented as median with interquartile range error bars. V̇O_2:_ oxygen consumption; mL: millilitre; kg: kilogram; min: minute; HR: heart rate; bpm: beats per minute

Physiological responses to exercise for control and experimental trials are presented in Fig. 2. No statistically significant differences were observed for V̇O_2_, HR or RPE between experimental and control trials (all *p* > 0.05), although there were some trends. Overall, V̇O_2_ was 4.5% higher in experimental trials (41.9 [5.3]) compared to control trials (40.1 [5.2]) with only a small effect (*r* = 0.24). In contrast, HR was slightly lower (0.4%) in control trials (161.5 [22.3]) compared to experimental trials (160.9 [19.1]) with a trivial effect (*r* = − 0.05). RPE was 7.7% higher in the experimental trials (14.0 [2.5]) when compared to control trials (13.0 [2.5]) with a small effect (*r* = 0.32).

**Fig. 3:**
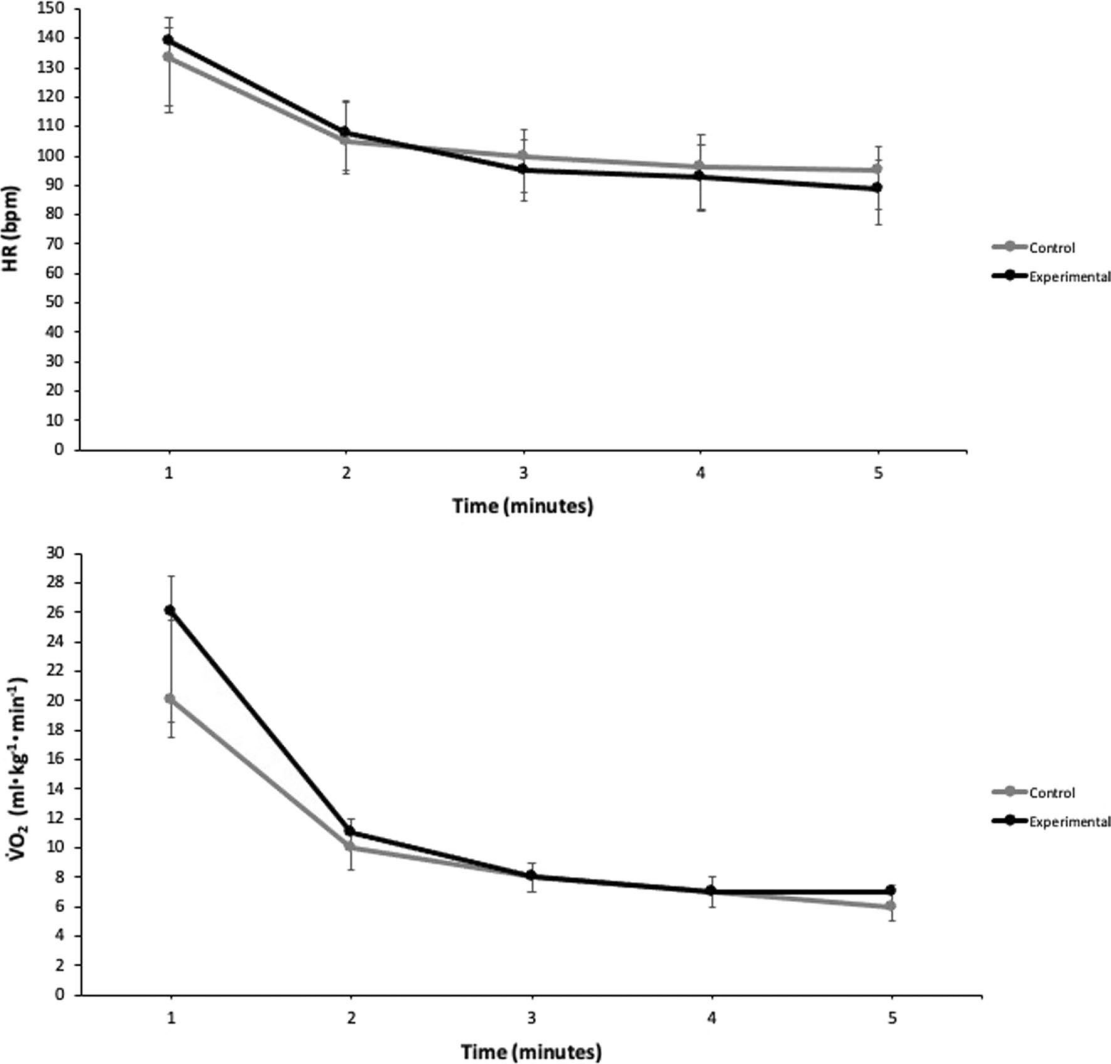
Physiological responses during five minutes post-exercise recovery in control and experimental trials (n = 15). Data presented as median with interquartile range error bars. V̇O_2:_ oxygen consumption; mL: millilitre; kg: kilogram; min: minute; HR: heart rate; bpm: beats per minute

Physiological responses elicited during post-exercise passive recovery for control and experimental trials can be viewed in Fig. 3. A significant effect of time was identified for V̇O_2_ and HR (*p* < 0.05) post-exercise, however no significant differences were observed for V̇O_2_ or HR between experimental and control trials (all *p* > 0.05). Some trends were identified—overall, V̇O_2_ was 4.7% lower in experimental trials (8.2 [2.1]) compared to control trials (8.6 [2.0]) with a small effect (*r* = − 0.32). Similar results were seen for HR with experimental trials (95.8 [21.3]) being 4.3% lower compared to control trials (100.1 [23.5]) with only a small effect (*r* = − 0.27).

### Individual responses during exercise and passive recovery

**Fig. 4:**
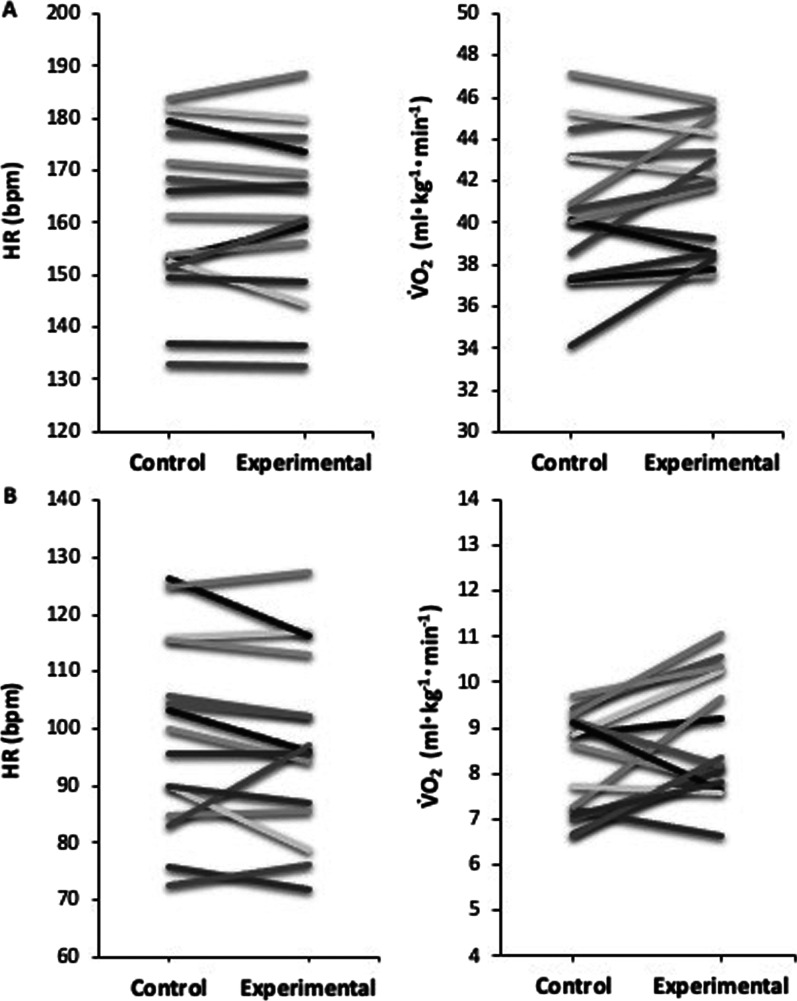
Individual responses during **(A)** five minutes steady-state running and **(B)** post-exercise recovery (n = 15). Results for control and experimental trials are presented as median per subject. V̇O_2:_ oxygen consumption; mL: millilitre; kg: kilogram; min: minute; HR: heart rate; bpm: beats per minute

Individual physiological responses to exercise in control and experimental trials are presented in Fig. 4A. V̇O_2_ was higher in experimental than control trials for 10 subjects (range: 0.4–12.0% higher) and lower in experimental than control trials for five subjects (range: 1.9–3.8% lower). The standard error for differences between experimental and control trials for V̇O_2_ was 1.2, and seven subjects showed a difference larger than this error. HR was higher in experimental than control trials for six subjects (range: 0.4–6.0% higher) and lower in experimental than control trials for nine subjects (range: 0.3–5.5% lower). The standard error for differences between experimental and control trials for HR was 5.8, and three subject showed a difference larger than this error. RPE was higher in experimental than control trials for five subjects (range: 6.7–16.7% higher), lower in experimental than control trials for one subject (6.7% lower), and unchanged for nine subjects. The standard error for differences between experimental and control trials for RPE was 0.8, and six subjects showed a difference larger than this error.

Individual physiological responses elicited during post-exercise passive recovery for control and experimental trials are also presented in Fig. 4B. Results showed that V̇O_2_ was higher in experimental than control trials for ten subjects (range: 4.4–33.4% higher) and lower in experimental than control trials for five subjects (range: 1.2–16.4% lower). The standard error for differences between experimental and control trials for V̇O_2_ was 0.4, and 13 subjects showed a difference larger than this error. Individual responses also showed that HR was higher in experimental than control trials for five subjects (range: 1.0–17.2% higher) and lower in experimental than control trials for nine subjects (range: 2.2–12.6% lower). One subject experienced no change in HR between control and experimental trials. The standard error for differences between experimental and control trials for HR was 6.0, and four subjects showed a difference larger than this error.

## Discussion

### Responses during exercise

During steady-state running, V̇O_2_ and RPE were higher (by 4.5% and 7.7%, respectively) and HR was 0.4% lower during experimental trials wearing the resistance garment than during control trials wearing a standard pair of exercise shorts, although the magnitudes of difference were trivial to small, and not statistically significant. Group-based results should be interpreted with caution as there was large inter-individual variability; V̇O_2_ was higher in experimental trials for 10 of the 15 subjects (range: 0.4–12.0% higher compared to control trials), HR was lower in experimental trials for nine of the 15 subjects (range: 0.3–5.5% lower compared to control trials), and RPE was unchanged between experimental and control trials for nine of the 15 subjects. Some subjects showed differences in V̇O_2_ (seven subjects), HR (three subjects) and RPE (six subjects) between experimental and control trials that were of greater magnitude than the standard error for differences between trials for the group. Taken together, these individual evaluations provide a different perspective and support an initial proof of concept despite non-statistically significant results. The V̇O_2_ response to exercise in this study is consistent with previously reported findings that lower limb loading increases the metabolic cost of exercise when compared to exercising in unloaded conditions [9–11]. Whilst the increase in V̇O_2_ during the experimental trials was of small effect in the current study (*r* = 0.24), the magnitude of this increase (4.5%) is greater than that reported by Martin [9] who attached 0.5 kg weights to the thighs and feet (1.7% and 3.3% respectively) and attached 1.0 kg weights to the thighs (3.5%) during continuous running. Others have reported similar increases in V̇O_2_ to the present study during submaximal running; Claremont and Hall [10] reported a 4.5% increase with the use of 0.45 kg ankle weights and Field et al. [11] reported an increase of 4.3% with the use of weighted pouches stitched into compression shorts equating to 3% BM resistance. Comparable increases in V̇O_2_ between the current study and previous research indicates the method of application of load to the garment in the current study (i.e. resistive bands woven into the material of the resistance garment) has similar potential to increase the metabolic demand as previously reported methods, with the added benefit of improved design features. However, caution should be used when generalising these findings as five of the 15 subjects in the current study recorded a lower V̇O_2_ in experimental trials (range: 1.9–3.8% lower compared to control trials), suggesting that the current WR garment may not influence the metabolic demand of running for everyone equally. Likely explanations for this may relate to differences in body morphology or the approach used to prescribe exercise intensity in the current study (i.e. assigning running speed based on physical activity levels vs. using a relative percentage of each subject’s V̇O_2_max). Nevertheless, there is a trend to suggest that the magnitude of applied resistance provided by the garment in the current study (1–3% BM) is likely sufficient to provide a stimulus that increases V̇O_2_ during a single bout of exercise. This finding supports those of Field et al. [11] who also observed 1.7%, 2.4% and 4.3% increases in V̇O_2_ when 1%, 2% and 3% BM resistance, respectively, were applied via lower limb loading during running. However, Field et al. [11] also observed 5.4% and 8.1% increases in V̇O_2_ when 4% and 5% BM resistance was applied, respectively, suggesting that V̇O_2_ may indeed continue to increase with increasing WR load. Therefore, future research is warranted to investigate an “upper limit” of WR that results in an increase in V̇O_2_ without a concomitant decrease in movement quality. Future research should also extend on the present investigation and assess whether the current WR garment elicits a similar V̇O_2_ response during exercise exceeding 10 min in duration and/or exceeding submaximal intensities that are individualized to each subject’s V̇O_2_max.

The perceived impact of the WR garment was insignificant, as shown by only a slightly higher RPE during the experimental (14 [2.5]) compared to the control trial (13 [2.5]). Two previous studies have examined the perceived impact of WR and external loading on the user during running and walking; both studies reported considerable increases in RPE when the % of BM resistance was increased using weighted back packs [8] and compression shorts [11]. Key methodological differences may explain, at least in part, why the present results do not align with those previously reported. The current study only assessed 1–3% BM resistance whereas Simpson et al. [8] assessed 20%, 30% and 40% BM resistance. In the current study, subjects completed 2 × 10-min steady-state runs with five days between trials whereas in the study by Field et al. [11], subjects completed 6 × 8-min submaximal runs over two separate testing sessions with only 2–3 days recovery between sessions. Additionally, it is unclear whether higher RPE scores in the Simpson et al. [8] study were the result of increases in BM resistance or a reflection of discomfort experienced by the subjects given the high discomfort ratings reported in the shoulders, neck, upper back, lower back, hips, thighs and lower legs. Although results from the current study do not support those from previous literature, the differences across studies highlights the need to further examine the perceived impact of WR during exercise using a variety of WR methods and applied magnitudes of resistance. Additionally, future research should also consider other perceptual measures that the current study did not explore such as comfort, to determine the practicality of WR methods more holistically.

In contrast to V̇O_2_, there was no difference in HR responses to exercise between the experimental and control trials. Indeed, HR was actually 0.4% lower in the experimental compared to control trials, although the effect size was trivial (*r* = − 0.05). This finding is unique when compared to previous literature as a variety of studies using lower limb loading [9–11] and upper body loading [8] noted an increase in HR when incorporating WR during exercise. Claremont and Hall [10] observed a 2.7% increase in HR when 0.45 kg ankle weights were used during continuous running. Martin [9] noted HR increases of 0.5% and 1.6% when 0.50 kg lead shot weights were attached to the thigh and feet respectively, and 1.4% and 3.4% HR increases with 1.0 kg lead shot weights attached to the aforementioned body segments. Field et al. [11] also reported increases in HR (0.4%, 1.5% and 1.8%) when 1%, 2% and 3% BM resistance was applied, respectively, via weighted pouches stitched onto compression shorts during sub-maximal running. Differences in the methods of WR used, and the loads applied between the current study and others may explain, at least in part, why different HR responses were seen. The general design of the garment in the present study and use of resistive bands may not provide consistent and/or sufficient load or resistance to influence HR during locomotion when compared to the addition of weighted pouches or lead shots used in previous research [9, 11]. However, further studies incorporating biomechanical analyses are needed before making conclusions regarding loading over a gait cycle. Additionally, the garments were not designed to specifically fit each subject’s individual body shape and size which may explain, at least in part, why nine of the fifteen subjects in the present study recorded a lower HR overall during experimental than control trials (range: 0.3–5.5% lower). The magnitude of HR increases in previous literature is lower than the magnitude of increase seen for V̇O_2_ [9–11], and this is consistent with the present study. This may suggest that V̇O_2_ is more sensitive to identifying a change in physiological load with WR, and therefore HR may have a higher threshold for detecting additional load applied in the form of WR garments. Future research examining the different detection limits of V̇O_2_ and HR over a range of resistances is needed to confirm this observation and identify their respective sensitivity thresholds.

### Responses during passive recovery

During passive recovery following steady-state running, V̇O_2_ and HR were lower (by 4.7% and 4.3%, respectively) during the experimental than the control trial and the magnitudes of difference were small and not statistically significant. V̇O_2_ was higher in experimental comparted to control trials for 10 of the fifteen subjects (range: 4.4–33.4% higher) and HR was lower in experimental compared to control trials for nine of the fifteen subjects (range: 2.2–12.6% lower), indicating large inter-individual variability in responses. Individual analyses also showed 13 of 15 subjects had differences in V̇O_2_ between trials that were larger than the standard error of the difference between trials. This was not consistent for HR where only four of 15 subjects showed a difference larger than the standard error of the difference between trials. Nevertheless, this variation in individual responses highlights the need for caution when generalising the main findings of the present study, especially given the small sample size. These results might hold some practical significance, since even small improvements in recovery could be valuable for exercise involving repeated efforts, such as high-intensity interval training where the intensity of the subsequent exercise bout is influenced by the preceding recovery bout [27, 28]. This is unique given the current study is the first to analyse the impact of lower limb WR on acute recovery following exercise as well as assessing a unique application of WR wholly contained within a garment and not using external weights. These findings provide a foundation for future research to investigate the influence of exercise intensity, duration and nature (continuous vs. intermittent) on recovery whilst wearing WR garments. The design of the garment used in the present study may explain, at least in part, why a small improvement in physiological recovery was seen during the experimental trial. The overall design and materials used ensured the garment smoothly conformed to the user’s individualized body shape creating a tight fit, similarly to a compression garment but with more specifically targeted resistance. Recent research has shown whole-body compression garments significantly reduced HR following incremental running tests in untrained subjects when compared to non-compression garments [29, 30]. When applied to garments, compression is proposed to improve recovery by mitigating the physiological strain of exercise via increased localized blood flow and provision of oxygen and improved venous return to remove metabolites following exercise [14]. While these mechanisms may partly explain the small reductions in V̇O_2_ and HR during passive recovery post-exercise, results should be interpreted with caution when determining the capacity of the garment to promote post-exercise recovery. These findings support the need for future research to further explore the impact of WR on recovery and the physiological mechanisms responsible. The measurement of additional metabolic variables such as blood lactate concentration may also be beneficial when exploring the extent to which WR increases the physiological demand of exercise, and its impact on post-exercise recovery.

### Study limitations

This study contains some limitations. Firstly, relative intensities for the ‘low’ and ‘high’ running speeds were determined via predictive equations as opposed to direct assessment of maximal exercise capacity. Given subjects were recreationally active adults, the stratification criteria of achieving either < 150 min/week or > 150 min/week of moderate intensity physical activity was adopted to assign running speeds as this cut off relates to the physical activity and exercise guidelines for Australian adults [17]. This criterion is also used in the ESSA-APSS when assigning risk factors for adverse events during exercise in which completing < 150 min/week is considered a risk factor. With seven subjects in this study self-reporting < 150 min/week of moderate intensity physical activity, it was deemed unsafe for subjects to complete a maximal test to determine individual exercise capacity. The garment in this study did not apply a customised level of resistance for each individual subject. However, subjects were encouraged to try on the three different sizes provided to ensure the garment worn during the trials smoothly conformed to their individual body shape. This was also to ensure the 'X’ shaped resistive bands shown in Fig. 1 were anatomically situated on each subject as similarly as possible.

## Conclusions

Although the effects were small, the novel WR garment increased V̇O_2_ and RPE during steady-state running, and reduced V̇O_2_ and HR during passive recovery, compared to control. With large inter-individual variability in results, conclusions can be drawn with some confidence that proof of concept is confirmed, suggesting that this garment design may be effective as a mechanism to increase training stimulus during running and as a recovery aid post-exercise, in recreationally-active men.

## Data Availability

The datasets used and/or analyzed during the current study are available from the corresponding author on reasonable request.

## Abbreviations

ACSM: American College of Sports Medicine
BM: Body mass
BMI: Body Mass Index
CO_2_: Carbon dioxide
FEA: Finite Element Analysis
HR: Heart rate
O_2_: Oxygen
RPE: Rate of perceived exertion
V̇CO_2_: Volume of exhaled cardon dioxide
V̇E: Ventilation volume
V̇O_2_: Oxygen consumption
V̇O_2_max: Maximal oxygen consumption
WR: Wearable resistance
